# Health risk assessment of particulate matter 2.5 in an academic metallurgy workshop

**DOI:** 10.1111/ina.13111

**Published:** 2022-09-18

**Authors:** Setlamorago Jackson Mbazima

**Affiliations:** ^1^ School of Geography, Archaeology and Environmental Studies University of the Witwatersrand Johannesburg South Africa; ^2^ Department of Environmental Sciences, College of Agriculture and Environmental Sciences University of South Africa Johannesburg South Africa; ^3^ Department of Toxicology and Biochemistry National Institute for Occupational Health Division of the National Health Laboratory Service Johannesburg South Africa

**Keywords:** carcinogenic, deposition, mass concentration, MPPD model, students, technicians

## Abstract

Exposure to indoor PM_2.5_ is associated with allergies, eye and skin irritation, lung cancer, and cardiopulmonary diseases. To control indoor PM_2.5_ and protect the health of occupants, exposure and health studies are necessary. In this study, exposure to PM_2.5_ released in an academic metallurgy workshop was assessed and a health risk assessment was conducted for male and female students and technicians. Polycarbonate membrane filters and an active pump operating at a flow rate of 2.5 L/min were used to collect PM_2.5_ from Monday to Friday for 3 months (August–October 2020) from 08:00–16:00. PM_2.5_ mass concentrations were obtained gravimetrically, and the Multiple‐Path Particle Dosimetry model was used to predict the deposition, retention, and clearance of PM_2.5_ in the respiratory tract system. The risk of developing carcinogenic and non‐carcinogenic effects among students and technicians was determined. The average PM_2.5_ mass concentration for August was 32.6 μg/m^3^ 32.8 μg/m^3^ for September, and 32.2 μg/m^3^ for October. The head region accounted for the highest deposition fraction (49.02%), followed by the pulmonary (35.75%) and tracheobronchial regions (15.26%). Approximately 0.55 mg of PM_2.5_ was still retained in the alveolar region 7 days after exposure. The HQ for male and female students was <1 while that of male and female technicians was >1, suggesting that technicians are at risk of developing non‐carcinogenic health effects compared with students. The results showed a risk of developing carcinogenic health effects among male and female technicians (>1 × 10^−5^); however, there was no excess cancer risk for students (<1 × 10^−6^). This study highlights the importance of exposure and health studies in academic micro‐environments such as metallurgy workshops which are often less researched, and exposure is underestimated. The results also indicated the need to implement control measures to protect the health of the occupants and ensure that the workshop rules are adhered to.

**Practical Implications^1^**

- This is the first exposure and health study on PM_2.5_ conducted in an academic metallurgy workshop in RSA.
- Using a method that considers the deposition fraction of PM_2.5_ in the human respiratory tract to conduct a health risk assessment is conservative and yields lower values compared with the conventional USEPA method.
- According to the health risk analysis, workshop technicians, particularly males are at a higher risk of developing carcinogenic health effects relative to students.

## 1 INTRODUCTION

Air pollution is a global environmental and public health concern, and particulate matter (PM_2.5_) has been identified as a leading contributor to poor outdoor and indoor air quality and a significant cause of cardiopulmonary disorders, lung cancer, hospitalization, and premature death.^2^, ^3^ According to the World Health Organization, air pollution is responsible for 7.3 million premature deaths and 4.3 million of these are attributed to poor indoor air quality.^2^ Exposure to PM_2.5_ in indoor micro‐environments is suggested to be more harmful relative to outdoor environments.^4^, ^5^ This is attributable to the confined nature of indoor environments that allows the accumulation, less dilution, transformation, and dispersion of PM_2.5._ ^6^, ^7^, ^8^ Furthermore, exposure per unit mass of PM_2.5_ released from indoor sources is between two to three orders of magnitude larger than in outdoor environments.^9^, ^10^ Studies^11^, ^12^ have found that the concentration of indoor PM_2.5_ tends to be higher than the outdoor in many cases. This is concerning given that in modern society people spend 90% of their time in confined indoor environments such as offices, classrooms, homes, and laboratories.^13^, ^14^

Buildings with natural ventilation mechanisms tend to have higher PM concentrations relative to mechanically ventilated buildings.^15^ This is because the mechanical ventilation system prevents the penetration of outdoor PM into indoor micro‐environments and also filters the indoor concentration.^16^ In the absence of significant sources, indoor PM_2.5_ concentration can be affected by PM penetrating from the outside.^17^ The penetration efficiency of PM_2.5_ into indoor micro‐environments depends on the structure of the building, infiltration rate and ventilation mechanisms.^18^ Room occupancy and movement also contribute significantly to the concentration of PM_2.5_ in micro‐environments.^19^ Chen et al.^12^ found that the concentration of PM_2.5_ in four laboratories at the National Pingtung University of Science and Technology increased by 4.9‐fold during classes and decreased significantly during recess. This also indicates that academic micro‐environments such as workshops are significant sources of PM_2.5_ and exposure is likely to occur.

Exposure to indoor PM_2.5_ is linked with allergies,^20^ eye, nose, throat, and skin irritation,^21^ coughing, sneezing,^22^ lung cancer,^23^ cardiovascular disorders, and respiratory diseases,^13^, ^24^ particularly among susceptible groups such as children, the elderly, and comorbid individuals.^13^, ^25^ Even at lower concentrations, exposure to PM_2.5_ in indoor micro‐environments can have adverse health effects among susceptible groups.^17^ Kim and Kang^26^ found that the deposition of PM is higher in individuals with comorbidities such as asthma and chronic obstructive pulmonary diseases (COPD). This is because asthma and COPD cause inflammation and narrowing of the airways.^27^ On the contrary, there is a linear relationship between the obstruction of airways and the deposition of PM, which leads to significant doses.^27^ Exposure can occur through dermal, ingestion, and inhalation route, however, the inhalation route has been specified as the most common and harmful route of entry.^28^, ^29^ This is because the olfactory nerves that bypass the blood–brain barrier are considered the shortest and direct pathway to the brain; therefore, particles can translocate directly into the brain.^27^ The toxicity of PM_2.5_ depends on the elemental composition, number concentration, particle shape and size,^27^, ^30^ whereas the severity of the health outcomes depends on the frequency and duration of exposure, concentration, individual characteristics, and route of entry.^31^, ^32^, ^33^

Exposure to PM_2.5_ can cause lung cancer irrespective of its composition; hence, the International Agency on Cancer Research has classified PM as a group one carcinogen.^34^, ^35^ Studies^36^, ^37^, ^38^ using predictive mathematical dosimetry models have shown that smaller particles penetrate deeper into the alveolar region and even translocate to vital organs while larger particles deposit in the upper respiratory tract region. Smaller particles have a large surface area per unit mass and can, therefore, occupy a larger surface area causing adverse health effects.^39^ Furthermore, particles that deposit deeper into the respiratory system are difficult to remove, consequently causing adverse health effects due to interactions with tissues and cells.^40^ Therefore, it is important to determine the uptake, redistribution, and clearance of particles in the human respiratory tract.^41^

Despite the risks associated with exposure to indoor PM_2.5_, indoor air quality is less researched relative to outdoor air quality—particularly in low‐middle‐income countries including the Republic of South Africa (RSA).^42^, ^43^ Specifically, indoor PM_2.5_ in academic micro‐environments such as metallurgy workshops where PM is likely to be generated has not been investigated in RSA. Consequently, there is a lack of literature on exposure and health in academic metallurgy workshops. Academic workshops are important for training and research; however, many workshops provide an enclosed and overpopulated micro‐environment characterized by many apparatuses and the frequent use of harmful substances.^44^ Moreover, students and staff can spend 7–9 h in such enclosed micro‐environments.^45^ Studies elsewhere^12^, ^15^, ^46^, ^47^, ^48^, ^49^, ^50^, ^51^, ^52^, ^53^ have investigated PM of different sizes in various academic micro‐environments and reported significant concentration levels. Despite this, exposure to PM_2.5_ in academic workshops in RSA is underestimated. Exposure to indoor PM_2.5_ in academic environments is of concern since students and technicians spend much of their time there and exposure rarely produces identifiable health outcomes until in the later stages of life.^54^ Moreover, poor indoor air quality is linked with discomfort, sick building syndrome, reduced memory, productivity, and performance among occupants.^55^, ^56^

To determine the degree of health risks associated with exposure to PM_2.5_ in indoor micro‐environments and possible mitigation strategies required to lower the exposure where necessary, exposure and health studies are important. The objectives of this study were to predict the deposition and retained dose and clearance of PM_2.5_ in the human respiratory tract system and to conduct a health risk assessment (HRA). This study presents the first exposure and health results in an academic metallurgy workshop in RSA. The findings from this study can help raise awareness about exposure to indoor PM_2.5_ since indoor air quality receives little attention in RSA. The findings might also assist policymakers to develop exposure control strategies in indoor micro‐environments at academic institutions.

## 2 METHODS AND MATERIALS

### 2.1 Site description

**FIGURE 1:**
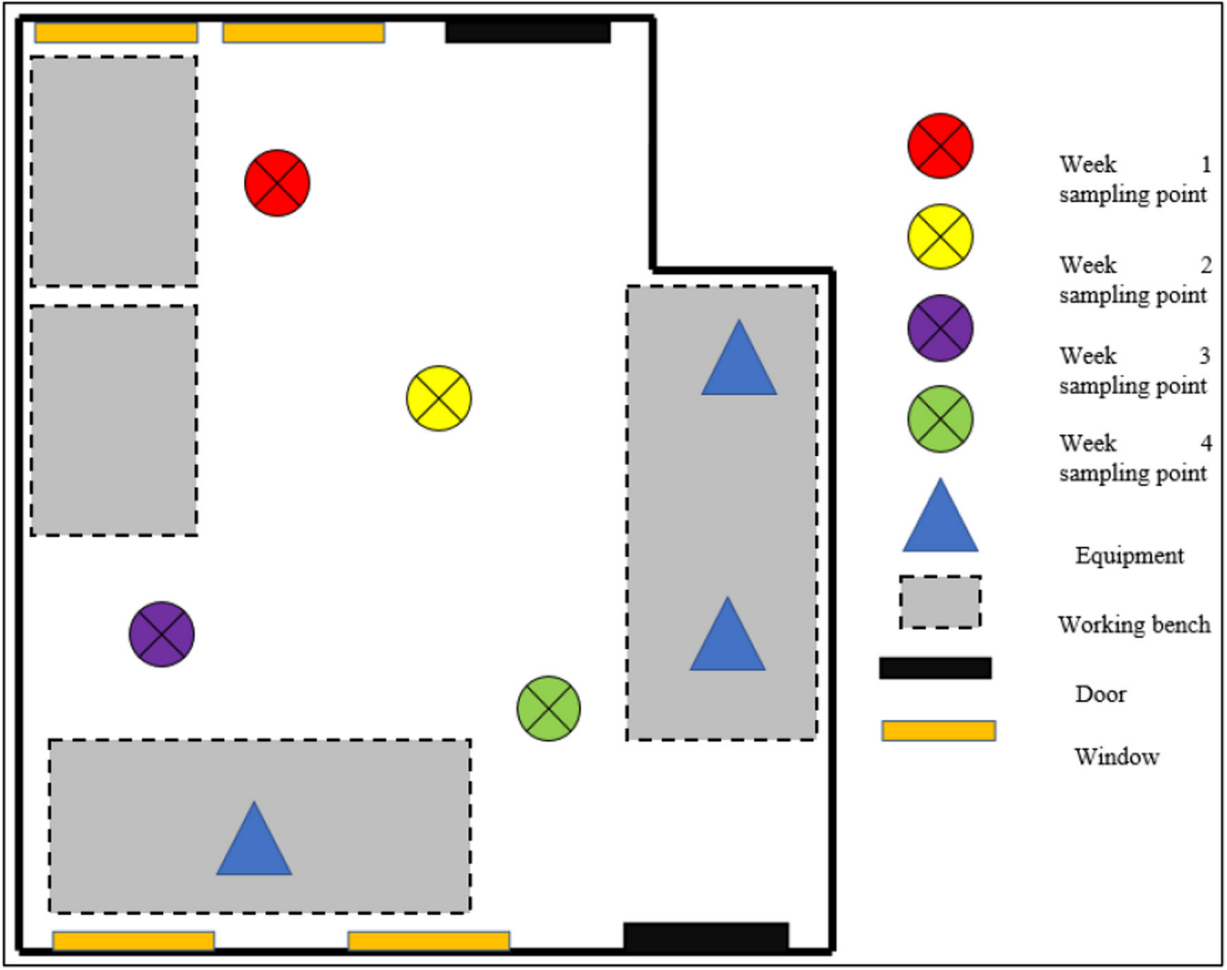
Layout of the metallurgy workshop and sampling points

The study was conducted in an academic metallurgy workshop used by students registered for the metallurgy and chemical engineering technology undergraduate degree, which is a 4‐year program. Activities in the workshop are facilitated by 3 technicians. A jaw crusher, roll crusher, and cone crusher are used to crush and reduce the size of specimens such as mineral‐enriched rocks or coal to smaller sizes. Minerals such as gold, platinum, chromite, copper, and silver are deemed valuable and therefore extracted. Silica is the least valuable mineral released during the process; hence, it is not extracted. Cement is also used in the process to add strength to the specimens under investigation. The processes release PM that is suspended in the air and settles over time. The settled PM is then re‐suspended by air movement and the movement of students while walking inside the workshop. The workshop is 7 × 5 m with 4 windows (Figure 1), however, the windows are never opened. Furthermore, the workshop does not have a mechanical ventilation system. As part of entering the workshop, personal protective clothing consisting of a laboratory coat, safety shoes, mask, and goggles are mandatory. However, during a walkthrough survey, it was noticed that students and technicians were not adhering to the rules of the workshop. Few students only wore laboratory coats as part of personal protective clothing.

### 2.2 Preparation of filters and sampling

Polycarbonate membrane (PCTE) filters (Zefon International Inc.) with pore sizes of 0.08 μm and diameter of 37 mm were conditioned for 48 h in a laboratory; temperature (21 ± 2°C), relative humidity (35% ± 3), and (dew point 6 ± 1%). An electronic microbalance scale (Sartorius, AG, model‐CPA225D) with a sensitivity of ±0.01 mg was used to pre‐weigh the filters. A Gilian GilAir 300 plus pump (Sensidyne) working at a constant flow rate of 2.75 L/min was used to collect samples for 3 months from 08:00–16:00. The pump was mounted on a tripod set at ~1.5 m to mimic the breathing height of a human being. The pump was connected to a 37 mm cassette (SKC) with a PCTE filter through a 0.5 m Teflon tube. The cassette was connected to a 37 mm PM_2.5_ Gs‐3 multiple‐inlet cyclone (SKC), which used centrifugal force to separate coarse and fine particles, with the fine particles deposited on the PCTE filter.

Gravimetric weighing was conducted using the same microbalance scale to obtain the post mass of the filter, each filter was weighed three times and the arithmetic average mass was taken. To obtain the final corrected mass, the difference between the pre‐ and post‐weights of the filter was calculated. The mass concentration was calculated using Equation (1).

(1) $$C=\frac{M}{V}$$

Where M is the final mass obtained gravimetrically, V is the volume obtained by multiplying the flowrate by the sampling time in minutes, divided by 1000 to convert from liters per minute (L/min) to cubic meters (m^3^).

### 2.3 Deposition

**FIGURE 2:**
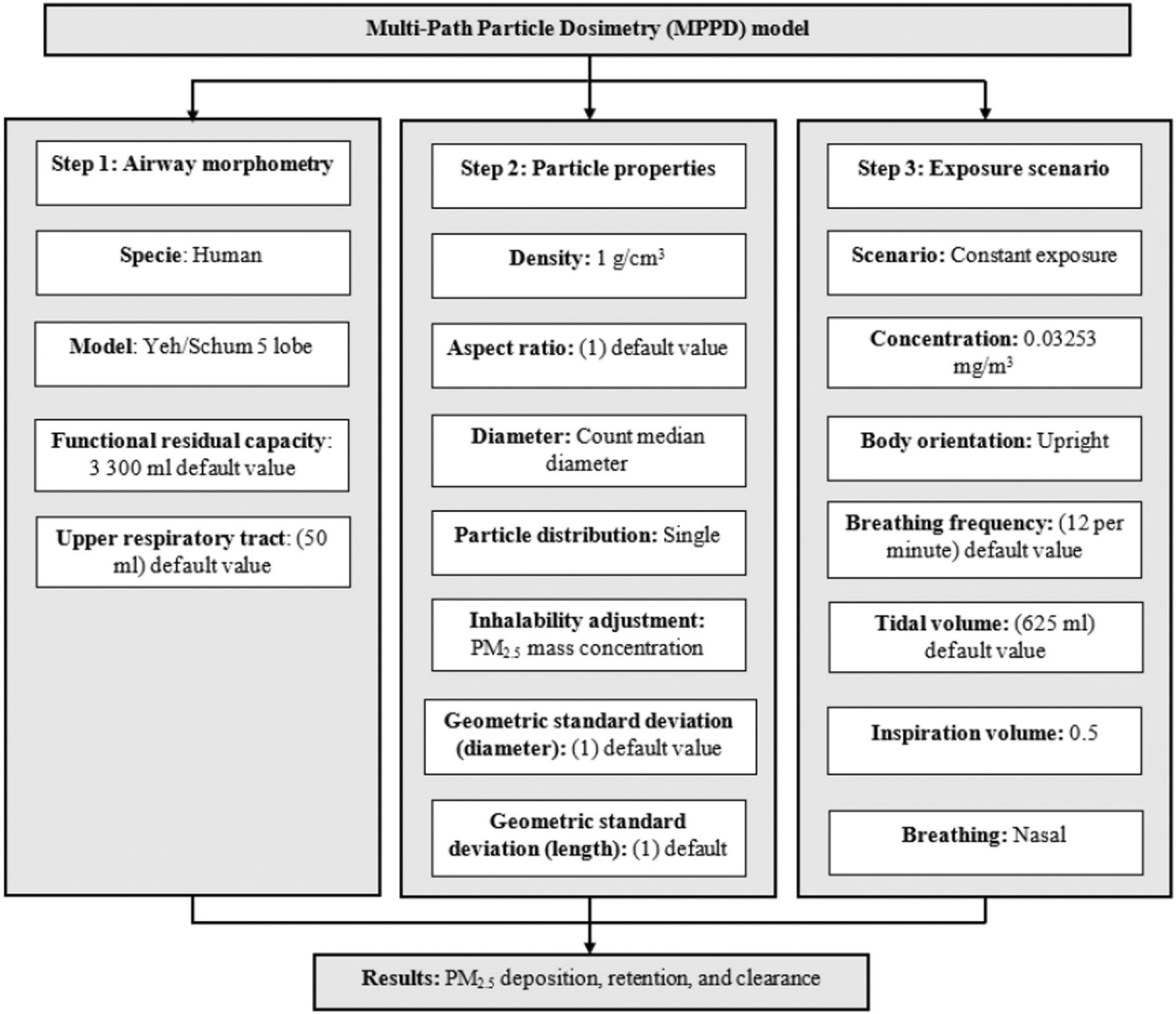
Framework and input parameters used in the multiple‐path dosimetry model

Multiple‐Path Particle Dosimetry (MPPD) is a mechanistic model that can be used to predict the deposition and clearance of monodisperse and polydisperse aerosols between 1 nm and 100 μm.^57^ The MPPD model was developed by the Hamner Institute for Health Sciences and the Dutch National Institute for Public Health and the Environment and is freely available from https://www.ara.com/mppd/.^58^ Version 3.04 of the MPPD model (Applied Research Associates Inc.) was used to predict the deposition, retention, and clearance of PM_2.5_ in the lungs of exposed students and technicians. The deposition fraction of PM_2.5_ was then used as DF in Equation (3) to calculate the average daily dose. The deposition into the respiratory tract system was determined using the average mass concentration of PM_2.5_ for 3 months (32.53 μg/m^3^). However, it is worth noting that the input PM_2.5_ average mass concentration used in the model was 0.03253 mg/m^3^ because the MPPD model dictates the input concentration to be in mg/m.3 Figure 2 shows the parameters used in the MPDD model. For the deposition and clearance inputs, the number of days per week was set at 8 h, the number of days per week was set at 5 days, and the number of weeks was set at one.^59^

### 2.4 Health risk assessment

A HRA is a tool that uses procedures and systematic approaches to assess the nature, severity, and probability of developing negative health effects due to exposure to chemical or biological stressors in the environment and manage the potential health threats.^60^, ^61^ The HRA framework has four steps, (i) hazard identification, (ii) dose–response assessment (toxicity), (iii) exposure assessment, and (iv) risk assessment.^62^ The risk associated with exposure to PM_2.5_ through the inhalation route was evaluated as prescribed by the United States Environmental Protection Agency (USEPA) guidelines.^63^

#### 2.4.1 Hazard identification

Existing literature on the health risk associated with exposure to PM_2.5_ through the inhalation route was reviewed. The International Cancer Agency classified PM as a group one carcinogen, while the USEPA listed PM_2.5_ as a criteria air pollutant.^64^, ^65^ PM_2.5_ is also ranked as the 13th leading cause of premature death globally by the World Health Organization.^66^, ^67^

#### 2.4.2 Exposure assessment

The metallurgy workshop is open Monday to Friday from 08:00–16:00. Although the students are registered for the 4‐year program, they only start using the workshop in the second year of their studies for experimental and research purposes. Therefore, their lifetime exposure duration was 3  years, assuming they would complete their studies in record time. A review of the workshop's 12 months register indicated that on average, students spent 5 h in the workshop. The 2019 academic calendar was reviewed to calculate the number of days students spent in the metallurgy workshop. Recess days, weekends, public holidays, and examination days were subtracted from the 2019 academic calendar, and it was determined that the students spent 115 days in the metallurgy workshop.

The metallurgy workshop is operated by male and female technicians who spend an average of 3 h in the workshop supervising students and preparing equipment and samples. The same 2019 academic calendar was reviewed to calculate the number of days that the workshop technicians spent in the workshop. Public holidays and holidays for staff members were subtracted, and it was discovered that the technicians spent 155 days in the metallurgy workshop. The lifetime exposure duration for the metallurgy workshop technicians was assumed to be 25 years, which is the minimum number of years an individual can work in RSA. A summary of the parameters used in this study is presented in Table 1.

**TABLE 1:** Summary of the values used for male and female students and technicians

Parameter	Description	Value	Unit	Source
C	Concentration	32.53	μg/m^3^	This study
IR	Inhalation rate	20	m^3^/day	^68^
ET	Exposure time	S = 5	hours/day	This study
		T = 3		
EF	Exposure frequency	S = 115	days/year	This study
		T = 155		
BW	Body weight	68.1	Kg	^69^
ED	Exposure duration	S = 3	days/year	This study
		T = 25		
A	Years in lifetime	M = 60	years	^70^
		F = 67		
IUR	Inhalation unit risk	0.008	μg/m^3^	^71^

Abbreviations: F, females; M, males; S, students; T, technicians.

The average daily dose through the inhalation route was calculated using two methods, in the first method, the ADD was calculated using Equation (2) adapted from the USEPA,^63^ and the second method used Equation (3) adapted from Lyu et al.^72^ and Chalvatzaki et al.^73^ The difference is that Equation (3) considers the PM_2.5_ deposition fraction obtained using the MPPD model while Equation (2) does not.

(2) $$\mathrm{ADD}=\frac{C\times \mathrm{CF}\times \mathrm{IR}\times \mathrm{ET}\times \mathrm{EF}\times \mathrm{ED}}{\mathrm{BW}\times \mathrm{AT}}$$

(3) $$\mathrm{ADD}=\left(C\times \mathrm{CF}\times \mathrm{IR}\times \mathrm{DF}\right)\times \frac{\mathrm{ET}\times \mathrm{EF}\times \mathrm{ED}}{\mathrm{BW}\times \mathrm{AT}}$$

Where C is the PM_2.5_ mass concentration (μg/m^3^), CF is the conversion factor, IR is the inhalation rate (m^3^/h), ET is the exposure duration, EF is the exposure frequency, ED is the exposure duration, BW is the body weight (Kg), AT is the average exposure (days/years), and DF is the deposition fraction obtained using the MPPD model. For carcinogenic health effects, AT = (life expectancy × 365 days × 24 h). Instead of using the LE of 70 years prescribed by the USEPA, the life expectancy for male and female technicians was obtained from Statistics South Africa. The life expectancy in RSA as per Statistics South Africa^70^ is 60 and 67 years for males and females, respectively. To further improve the accuracy of the HRA, body weight values were obtained from a local study.^69^ The ADD for 3 and 25 years was calculated using Equation (4).

(4) $${\mathrm{ADD}}_{\text{cumulative}}=\sum \frac{\mathrm{ADD}\times 365\times \mathrm{ED}}{\mathrm{LE}}$$

where ADD is the average daily dose (mg/kg) calculated using Equations (2) and (3), 365 is the number of days in a year, ED is the exposure duration, and LE is the life expectancy in years. The adjusted average daily dose, which is 3 years for students and 25 years for technicians was calculated using Equation (5).

(5) $${\mathrm{ADD}}_{\text{adjusted}}=\frac{{\mathrm{ADD}}_{\text{cumulative}}}{\mathrm{LE}}$$

where ADD_cumulative_ is the average daily for the specific number of years for students and technicians obtained using Equation (4) and LE is the life expectancy in days.

#### 2.4.3 Risk characterization

Similar to de Oliveira et al.^74^ the diesel particulate reference concentration (RfC) of 5 μg/m^3^ was used to calculate the reference concentration dose (RfD) for PM_2.5_ due to a lack of consensus concerning the RfC. The RfD for PM_2.5_ was calculated using Equation (6).

(6) $$\mathrm{RFD}=\frac{5}{1000\;\left(\mathrm{\mu g}/\mathrm{mg}\right)}\times \frac{\mathrm{IR}}{\mathrm{BW}}$$

where 5 is the RFC for diesel particulates, IR is the inhalation rate, and BW is the body. The hazard quotient (HQ) was then calculated using Equation (7) to determine the risk of developing non‐carcinogenic health effects among students and technicians at the metallurgy workshop.

(7) $$\mathrm{HQ}=\frac{{\mathrm{ADD}}_{\text{adjusted}}}{\mathrm{RfD}}$$

where ADD_adjusted_ is the adjusted average daily dose calculated using Equation (5) and RfD is the reference dose for diesel particulates calculated using Equation (6). A HQ greater than one indicated that students and technicians are at risk of developing adverse health effects while a risk quotient less than one indicates less risk. The risk of developing carcinogenic health effects due to exposure to PM_2.5_ through the inhalation route was also calculated. However, to calculate the cancer risk, the slope factor is needed, therefore, the slope for PM_2.5_ was calculated using Equation (8)

(8) $$\mathrm{SF}=\frac{\mathrm{IUR}}{\mathrm{BW}\times \mathrm{IR}}$$

Where IUR is the unit risk of PM_2.5_ adapted from Pope III et al.^71^ BW is the body weight, and IR in the inhalation rate. The risk of developing cancer (CR) among male and female students and technicians was then calculated using Equation (9).

(9) $$\mathrm{CR}={\mathrm{ADD}}_{\text{adjusted}}\times \mathrm{SF}$$

where ADD_adjusted_ is the adjusted average daily dose obtained using Equation (5) and SF is the slope factor calculated using Equation (8). The cancer risk is represented by the acceptable number of cancer cases in a population and the widely used scale of risks is 1 in million (1 × 10^−6^), 1 in one hundred thousand (1 × 10^−5^), and 1 in ten thousand (1 × 10^−4^).^75^, ^76^ A value greater than 1 × 10^−4^ indicates a significant cancer risk while a value less than 1 × 10^−6^ indicates an insignificant cancer risk that can be ignored.^77^, ^78^

### 2.5 Quality control

To calibrate the microbalance scale and validate the results, standard pendulums weighing 100 and 200 g were weighed on the microbalance before and after sampling. To avoid cross‐contamination, Teflon‐coated tweezers were used to handle the filters. Before and after weighing, the filters were conditioned for 24 h in a laboratory. Each filter was weighed three times and the average mass of the triplicate was used. Before sampling, background measurements were collected for 15 min using the same pump, and the results were subtracted from the actual sampling results. For each sampling session, a blank filter was prepared and placed next to the field filters during sampling and transportation to account for moisture loss owing to weather conditions. The filters were transported using a suitcase designed to keep both the filter and cyclone upright to prevent the loss of particles. A bubble flow meter (Sensidyne, St Petersburg, FL, USA) was used to verify the flow rate before and after sampling, and the fluctuation was less than 5%.

## 3 RESULTS AND DISCUSSION

### 3.1 PM_2_ _.5_ mass concentration

The PM_2.5_ mass concentration results for the 3 month sampling period in the academic metallurgy workshop are presented in Table 2. The lowest PM_2.5_ mass concentration (26.54 μg/m^3^) was recorded in the fourth week of August, while the highest (44.8 μg/m^3^) was recorded in the second week of August. Overall, the arithmetic average PM_2.5_ mass concentration in the metallurgy workshop during the 3‐month sampling period was 32.53 ± 3.04 μg/m^3^. Indoor air quality is not regulated in RSA, however, the USEPA recommends safe levels of 15 μg/m^3^ for indoor PM_2.5_ mass concentration. This means that the 3 months arithmetic PM_2.5_ mass concentration in the metallurgy workshop exceeded the USEPA recommended safe level by 2.2‐fold.

**TABLE 2:** PM_2.5_ mass concentration (μg/m^3^) at the metallurgy workshop

							CI (95%)	
Sampling campaign							Lower	Upper
Month	Week	Min	Mean	Max	SD	Monthly average	quartile	quartile
August	Week one	32.54	32.97	33.72	0.46	32.59	32.70	33.07
	Week two	33.03	38.19	44.80	4.42		35.74	39.49
	Week three	27.45	30.40	34.64	3.07		28.05	32.50
	Week four	26.54	28.82	31.34	1.96		27.31	30.07
September	Week one	28.64	31.05	33.17	1.77	32.83	30.14	32.23
	Week two	29.49	33.39	37.16	3.36		30.51	36.09
	Week three	29.74	34.00	36.46	3.06		30.77	36.46
	Week four	29.55	32.87	36.29	2.68		31.13	34.57
October	Week one	27.95	31.07	32.95	2.29	32.16	29.30	32.60
	Week two	26.84	32.55	37.16	4.39		36.02	36.02
	Week three	29.55	33.89	36.62	3.31		31.14	36.61
	Week four	30.06	31.14	32.91	1.22		30.32	31.91

Abbreviations: CI, confidence interval; Max, maximum; Min, minimum; SD, standard deviation.

Although the 3‐month average PM_2.5_ mass concentration was higher than the USEPA recommended level, it was lower compared to what previous studies reported. Gemenetzis et al.^48^ reported a PM_2.5_ arithmetic mass concentration of 91 ± 56 μg/m^3^ in a Chemical Department at the Aristotle University of Thessaloniki, Greece. Sahu et al.^50^ reported an average PM_2.5_ mass concentration of 38 μg/m^3^ in the air and noise pollution laboratory at the Indian School of Mines Dhanbad Campus, India. In another study conducted in dental prosthesis laboratories in Kocaeli, Turkey, Yıldırım et al.^47^ reported an arithmetic average PM_2.5_ mass concentration of 414 ± 406 μg/m^3^. Kumar and Jain^52^ recently reported an average PM_2.5_ mass concentration of 47.38 ± 2.64 μg/m^3^. Nonetheless, other studies have reported average PM_2.5_ mass concentrations lower than this study. Ugranli et al.^53^ conducted an indoor air quality study in the Department of Chemistry and Department of Chemical Engineering laboratories at Izmir Institute of Technology. Average PM_2.5_ mass concentrations in the chemistry laboratories were 9.3 ± 3.16, 18.7 ± 8.58, and 26.2 ± 10.4 μg/m^3^ while mass concentrations in the chemical engineering laboratories were 7.64 ± 8.86, 10.4 ± 4.77, and 19.4 ± 4.92 μg/m^3^. Recently, Bhat et al.^45^ reported a PM_2.5_ mass concentration of 28 μg/m^3^ in a material testing laboratory at the Eskişehir Technical University Campus in Eskişehir, Turkey.

### 3.2 PM_2_ _.5_ deposition

**FIGURE 3:**
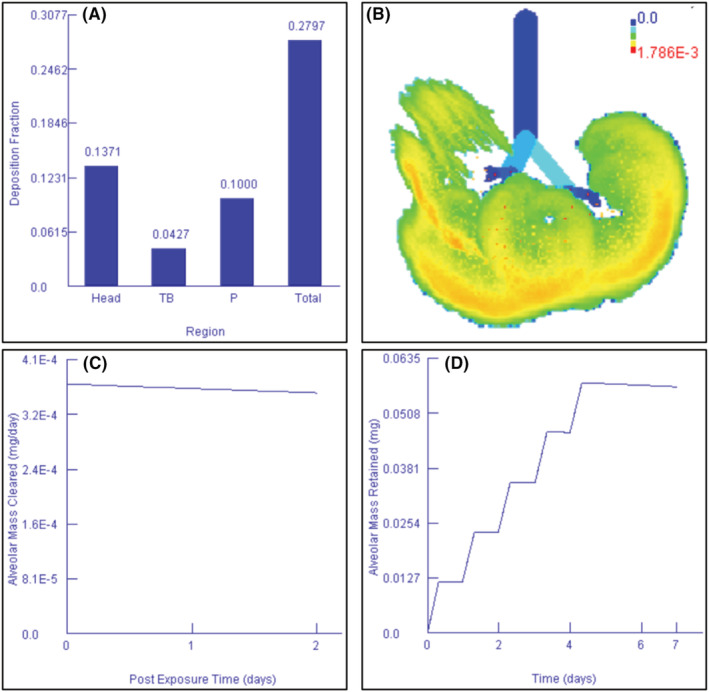
Predicted deposition of PM_2.5_ based on a 3‐month average concentration; (A) deposition of PM_2.5_ into different regions of the respiratory tract system, (B) visualized deposition of PM_2.5_ into the alveolar region, (C) clearance of PM_2.5_ in the alveolar region

The deposition, clearance, and retention of PM_2.5_ into the respiratory tract of exposed students and technicians simulated using the MPPD model are shown in Figures 3A–D. According to the MPPD model simulation results, exposure to a concentration of 0.03253 mg/m^3^ (32.53 μg/m^3^) will lead to a deposition fraction of 0.2797 in the respiratory tract system. From Figure 3A, it can be observed that the head region (49.02%) accounted for the highest deposition fraction, followed by the pulmonary region (35.75%) and tracheobronchial region (15.26%). Manojkumar et al.^79^ used the MPPD model to predict the deposition of PM in human airways and found that 45% of PM_2.5_ were deposited in the head region, 45% in the pulmonary region, and 9% in the tracheobronchial region. Madureira et al.^80^ predicted the deposition of PM_2.5_ in newborns and mothers and found that most particles were deposited in the head region, followed by the tracheobronchial region, whereas the least particles were deposited in the pulmonary region. In another study, Manojkumar et al.^1^ also found a similar trend whereby PM_2.5_ deposition in the respiratory tract was in the decreasing order of head > pulmonary > alveolar region.

Other studies have used different models but reported similar results. Martins et al.^81^ used the Exposure Dose Model to predict the deposition of PM_2.5_ and found that 68% deposited in the head region, 10% in the tracheobronchial region, and 4% in the pulmonary region. Although these studies used models different from the MPPD, the results showed a similar trend whereby a higher fraction of PM was deposited in the head region, followed by the tracheobronchial region and pulmonary region. Therefore, it can be concluded that regardless of the model used, the head region always accounts for most of the deposition of PM, followed by the tracheobronchial region and pulmonary region. The higher deposition of PM_2.5_ in the head region can be attributed to the longer residence time in the nasal cavity that allows particles to deposit.^27^

Figure 3B shows the visualization of the PM_2.5_ deposited in the alveolar region, and it can be observed that 0.00176 was deposited in the alveolar region. The deposited particles were probably of a smaller size, previous studies^17^, ^39^, ^82^, ^83^, ^84^ have shown that smaller particles tend to deposit in the alveolar region where they can interact with epithelial cells and macrophages—subsequently causing inflammation. Furthermore, smaller particles can cross the air‐blood barrier in the alveolar and translocate to the cardiovascular system.^85^

In Figure 3D, the clearance of PM_2.5_ in mg/day in the alveolar region is shown and it can be observed that the clearance occurred slowly. After 2 days of exposure, approximately 0.00032 mg was cleared in the alveolar region. From Figure 3D, it can be observed that after 7 days of exposure to PM_2.5_ concentration of 0.03253 mg/m^3^, approximately 0.55 mg was retained in the alveolar region. The findings agree with previous studies^79^, ^86^, ^87^ that reported the clearance of smaller particles in the alveolar region happens at a slower rate hence they can be retained longer. It has also been stated that the clearance of particles in the lower respiratory tract is slower than the upper respiratory tract. The airway surfaces in the head and tracheobronchial regions are lined with a layer of mucus propelled by the ciliary action to the gastrointestinal tract.^88^ Conversely, the alveolar region does not have a protective layer of mucus, hence the clearance of particles is slower, which poses significant health risks because gaseous exchange takes place in the alveolar region.^27^, ^88^

The slow clearance of particles in the alveolar region can be attributed to two natural mechanisms. The first is the absorptive mechanism whereby the PM is removed either by lymphatic transport or blood uptake, and the second is the non‐absorptive mechanism in which phagocytosis and macrophages are involved.^79^ These mechanisms are extremely slow; hence, the clearance can take days to months whilst other fractions of PM translocate to vital organs.^79^ Furthermore, the alveolar region has a long residence time, and the alveolar sacs and alveoli are tiny, hence, only a small fraction of the particles can be exhaled.

### 3.3 Health risk analysis

Table 3 presents the risk of developing non‐carcinogenic health effects among students and technicians obtained using Equations (2) and (3). Notably, the ADD and ADD_adjusted_ were higher for technicians than for students when using both equations. However, when using Equation (2), the ADD and ADD_adjusted_ for both male and female students and technicians were higher than when using Equation (3) that accounted for the deposition of PM_2.5_ particles into the respiratory tract. When using Equation (2), the ADD for male and female students and technicians was 3.6‐fold greater than when using Equation (3). The ADD_adjusted_ for male and female students was 3.6‐fold greater when using Equation (2) than (3); while the ADD_adjusted_ for male and female technicians was 8.4‐fold greater than when using Equation (3).

**TABLE 3:** Predicted intake dose and non‐carcinogenic risk results for male and female students and technicians

Receptors	Sex	Equation 2, ^63^			Equation 3, ^72^, ^73^		
		ADD	ADD_adjusted_	HQ	ADD	ADD_adjusted_	HQ
Students	Male	0.2747	0.0002	0.1559	0.0768	0.0001	0.0137
	Female	0.2460	0.0002	0.1119	0.0688	0.0001	0.0099
Technicians	Male	1.8510	0.0129	**8.7537**	0.5177	0.0015	**1.0397**
	Female	1.6576	0.0092	**6.2867**	0.4636	0.0011	0.7467

*Note*: Bold values show the risk of developing non‐carcinogenic health effects.

Similarly, the HQ for male and female students and technicians was higher when using Equation (2) than Equation (3) as shown in Table 3. However, the HQ for male students was 1.39‐fold greater than that of female students when using both Equations. When using Equation (2), the HQ of male and female students was 11.3‐fold greater than Equation (3); nonetheless, the HQ was less than one when using both equations, implying that the students were not at risk of developing non‐carcinogenic health outcomes. The HQ for male and female technicians was 2.4‐fold greater when using Equation (2) compared with Equation (3). The HQ of male and female technicians was higher than one when using Equation (2) meaning that they were at risk of developing non‐carcinogenic health outcomes because of exposure to PM_2.5_. Furthermore, the HQ of male technicians was 1.39‐fold greater than that of female technicians, implying that male technicians were at a higher risk of developing non‐carcinogenic health outcomes. Güneş et al.^89^ investigated indoor air quality in library universities and found a HQ less than one for students and library staff when using the USEPA equation. In this study, only the HQ for students was less than one when using the USEPA equation. The difference in the results might be partly explained by the different methodological approaches. The authors^89^ used the limit values for indoor PM_2.5_ prescribed by the World Health Organization as the RfC, whereas in this study, the RfC for diesel particulates was used to calculate the RfC for PM_2.5_.

Based on Equation (3), the HQ of male technicians was greater than one, but that of female technicians was less than one; indicating that male technicians are at risk of developing non‐carcinogenic health outcomes while female technicians were not. The findings showed that despite being exposed to the same concentration of PM_2.5_ and having the same deposition fraction, male technicians are at a higher risk of developing non‐carcinogenic health outcomes compared to female technicians. Using Equation (2) yielded higher estimates relative to Equation (3) due to particle deposition mechanisms, namely, impaction, sedimentation, and diffusion.^73^, ^90^ Therefore, it is not surprising that in this study, Equation (3) which accounted for particle deposition yielded lower estimates relative to Equation (2). The results of this study are consistent with Chalvatzaki et al.^73^ who also found lower estimates when using Equation (3) and concluded that the equation preserves values lower than one when calculating the ADD.

Table 4 shows the risk of developing carcinogenic health outcomes because of exposure to PM_2.5_ calculated using Equations (2) and (3). The cancer risk of male and female students was acceptable and insignificant (<1 × 10^−6^) when using Equation (2), while no excess cancer risk was found when using Equation (3). Based on Equation (2), the tolerable cancer risk of 1 × 10^−5^ was exceeded for both male and female technicians. It was found that two technicians in 100 000 were at risk of developing cancer. Although the tolerable cancer risk was exceeded, it was below 1 × 10^−4^ which is regarded as a significant cancer risk. No excess cancer risk was found for either male or female technicians when using Equation (3). Kim et al.^91^ conducted a risk assessment of PM_2.5_ in kitchens and living rooms using a similar approach as Equation 2 and found that the occupants were at risk of developing carcinogenic health effects (>1 × 10^−5^). A study by Heydari et al.^92^ conducted in a waterpipe café found that occupants were also at risk of developing carcinogenic health effects (>1 × 10^−5^) because of exposure to indoor PM_2.5._ Vo et al.^93^ found cancer risk due to exposure to PM_2.5_ through the inhalation route ranging from 2.3 × 10^−6^ to 4.9 × 10^−5^ in three age groups in residential indoor micro‐environments in Hanoi. Although the studies were conducted in different indoor micro‐environments, in general, the findings are in line with the existing and growing body of knowledge regarding the toxicity of PM_2.5_. The findings imply that control measures must be put in place to protect the health of occupants in indoor micro‐environments, particularly in metallurgy workshops where the release of PM_2.5_ is anticipated, and exposure is probable.

**TABLE 4:** Carcinogenic health risk assessment results of students and technicians

Receptors	Sex	Equation 2, ^63^	Equation 3, ^72^, ^73^
		Cancer risk	Cancer risk
Students	Male	4 × 10^−7^	10 × 10^−9^
	Female	3 × 10^−7^	7 × 10^−10^
Technicians	Male	**2 × 10** ^**−5**^	9 × 10^−8^
	Female	**2 × 10** ^**−5**^	6 × 10^−8^

*Note*: Bold values show a significant and unacceptable risk of developing cancer.

Like the HQ, the cancer risk was slightly higher when using Equation (2) than when using Equation (3), which accounted for the deposition fraction of PM_2.5_ in the respiratory tract system. Although there was no excess cancer risk for male and female students and technicians, there is no safe level of carcinogen exposure below which there is no likelihood of having carcinogenic health effects throughout an individual's lifetime.^94^ Therefore, it is recommended to implement control measures such as the wearing of masks while in the workshop since the cancer risk limits are for regulatory purposes.^46^, ^95^

### 3.4 Strengths and limitations

This is the first exposure and health study conducted during experimental activities in an academic metallurgy workshop in RSA. Instead of relying only on the traditional HRA method recommended by the USEPA, the MPPD model was used to predict the deposition and retention of the PM_2.5_ particles in the respiratory tract. The deposition fraction of PM_2.5_ into the respiratory tract was then used to predict the ADD and a HRA was conducted for both male and female technicians since they are affected in different ways.

This study also had limitations. The sampling duration was short; therefore, the findings must be taken as a snapshot of what is happening at the metallurgy workshop. The ventilation and air exchange rates, which are important when conducting indoor air quality, were not measured because of challenges with monitoring equipment. It was assumed that the exposure duration for students was 3 years. However, some students lag in their studies and spend more than 3 years doing experiments in the metallurgy workshop. Personal measurements obtained from the breathing zone are preferred in exposure assessments, particularly for workers. However, in this study, area measurements were used instead of personal measurements, which is a limitation that can underestimate the exposure. The assumptions and input parameters used in the MPPD model might not accurately represent students and technicians as well as susceptible individuals with compromised respiratory tract systems. The results must be interpreted with caution since some of the default values were obtained from the USEPA, which might not be a true reflection of the RSA population. Although the body weight used is from a local study,^69^ the which was conducted in a different province within RSA and might not accurately represent the body weights of the students and technicians where this study was conducted. Although the body weight might not accurately represent the students and technicians, it is a better representation compared to the 70 Kg recommended by the USEPA.

For improvement, future studies must consider taking personal measurements and air exchange rates and measuring for longer periods to get a better representation.^96^ Future studies should also obtain a measurement from the breathing zone and consider using questionnaires to document health effects among occupants and conduct spirometry tests, particularly on workshop technicians. Task‐based sampling must be conducted to check which equipment or processes emit significant concentrations of PM_2.5_ so that they can be prioritized when implementing control measures. The elemental composition and morphology of the particles in academic metallurgy workshops must be investigated since the health effects of exposure to PM depend on the size and chemical composition of the particles.

## 4 CONCLUSION

Exposure to indoor PM_2.5_ is a global public health concern because of the associated adverse health effects. This study assessed exposure to indoor PM_2.5_ for 3 months in an academic metallurgy workshop and conducted a HRA using two equations. The head region accounted for most of the deposited PM_2.5_ and particles deposited in the alveolar region were still retained 7 days after exposure. Equation (3), which accounted for the deposition fraction of PM_2.5_ into the human respiratory tract, yielded lower results relative to the USEPA equation that did not account for the deposition rate. Compared with students, technicians were at a higher risk of developing non‐carcinogenic health effects. However, male technicians were at a higher risk relative to females. Male and female technicians were also at risk of developing carcinogenic health effects however, there was no risk of developing carcinogenic health effects in students. Control measures must be implemented, and workshop rules must be followed to protect the health of occupants. Despite the limitations, this is the first study to provide insight into exposure to indoor PM_2.5_ in an academic metallurgy workshop in RSA and gives direction for future research in academic metallurgy workshops.

## AUTHOR CONTRIBUTIONS

The author was solely responsible for the conceptualization, methodology, data collection, data curation, data analysis, software, visualization, and writing, reviewing and editing the first and final draft of the manuscript.

## CONFLICT OF INTEREST

The author has no conflict of interest.

## Data Availability

The data that support the findings of this study are available on request from the corresponding author. The data are not publicly available due to privacy or ethical restrictions.
